# Interventions, Participative Role, Barriers, and Facilitators for Involvement in eHealth Communication for People Undergoing Hemodialysis: Protocol for a Scoping Review

**DOI:** 10.2196/38615

**Published:** 2022-07-29

**Authors:** Anne Deinboll, Cathrine Fredriksen Moe, Mette Spliid Ludvigsen

**Affiliations:** 1 Faculty of Nursing and Health Sciences Nord University Mo i Rana Norway; 2 Faculty of Nursing and Health Sciences Nord University Bodo Norway; 3 Department of Clinical Medicine Randers Regional Hospital Aarhus University Aarhus Denmark; 4 Danish Centre of Clinical Guidelines and Danish Centre of Systematic Reviews A Joanna Briggs Institute Centre of Excellence Aalborg University Aalborg Denmark

**Keywords:** chronic disease, decision-making, shared, electronic health records, nephrology, patient advocacy, patient portals, referral and consultation, renal dialysis, renal insufficiency, chronic, patient participation

## Abstract

**Background:**

eHealth interventions have been shown to offer people living with chronic kidney disease the opportunity of embracing dialysis therapies with greater confidence, the potential to obtain better clinical outcomes and increased quality of life, and diverse and flexible designs and delivery options. eHealth interventions or solutions can offer one-way information without the possibility for dialogue, as with most mobile apps. eHealth interventions intending to enable two-way communication between patients undergoing hemodialysis and health professionals are the focus of this review. eHealth communication interventions that enable two-way communication between patients undergoing hemodialysis and health professionals is an emerging field, but issues relating to participation in eHealth communication for patients undergoing hemodialysis are scarcely described. The current conceptualization of this issue is too scattered to inform the development of future interventions. In this scoping review, we want to assemble and examine this scattered knowledge on participation in two-way eHealth communication for patients undergoing hemodialysis.

**Objective:**

We want to understand the participative role of people living with chronic kidney disease undergoing hemodialysis in available communicative eHealth interventions and to understand which barriers and facilitators exist for patient involvement in eHealth communication with health professionals.

**Methods:**

A scoping review methodology is guiding this study. Peer-reviewed primary studies, including quantitative, qualitative, and mixed methods study designs will be included. A systematic search for published studies, dissertations, and theses at the doctoral level in the English language will be conducted in five databases (MEDLINE, Embase, CINAHL, Scopus, and ProQuest Dissertations and Theses). The included literature will focus on adult (18 years or older) patients undergoing hemodialysis who are involved in eHealth communication with health professionals. Data on the type of eHealth communication interventions, the participative role, and barriers and facilitators for the involvement in eHealth communication for people undergoing hemodialysis will be extracted independently by two reviewers. The extracted data will be collected in a draft charting table prepared for the study. Any discrepancies between the reviewers will be solved through discussion or with a third reviewer.

**Results:**

Results are anticipated by the spring of 2023 and will be presented in tabular format along with a narrative summary. The anticipated results will be presented in alignment with the objectives of the study, presenting findings on the participative role of patients undergoing hemodialysis in eHealth communication interventions.

**Conclusions:**

We anticipate that this study will inform on eHealth communication interventions and the level of patient participation in eHealth communication for patients undergoing hemodialysis. The systematized overview will possibly identify research gaps and motivate further development of eHealth communication to ensure patient participation. The findings will be of interest to key stakeholders in clinical care, research, development, policy, and patient advocacy.

**International Registered Report Identifier (IRRID):**

PRR1-10.2196/38615

## Introduction

### Background

Globally, people living with chronic diseases form a large and expanding group. The number of individuals worldwide with all-stage chronic kidney disease (CKD) reached almost 700 million in 2017 [1]. In 2020, kidney disease has risen from the world’s 13th leading cause of death to the 10th [2]. CKD is divided into five stages related to the glomerular filtration rate (GFR). A person with the most advanced stage of kidney disease, CKD5, has a GFR of 15 ml per minute or less, and kidney replacement therapy is needed. At this severe irreversible stage of kidney disease, the kidneys have lost nearly all of their ability to function effectively. The situation is associated with high mortality and comorbidity, including cardiovascular complications, diabetes, anxiety, depression, dietary and fluid restrictions, a comprehensive medication regime, social limitations to prevent infections, fatigue, suicidal ideation, and sexual dysfunction. The patients have an overall ill feeling related to the level of toxicity [3]. The numerous symptoms of CKD5 and the resulting regime of living required to limit the severity of the situation affect the quality of life (QOL) [4].

An estimated three million people with CKD5 receive kidney replacement therapy, either transplantation or various forms of dialysis [1]. There are two forms of long-term dialysis, both advanced and time- and cost-consuming. Peritoneal dialysis (PD) involves cleansing the blood inside the body using the peritoneal membrane and gaining access to the peritoneum through a catheter in the abdomen [5]. However, in-center hemodialysis is the dominant form of dialysis. Hemodialysis is when blood is pumped out of the body to an artificial kidney machine and returned to the body by tubes connecting the patient to the machine [6]. Hemodialysis involves direct access to the patients’ circulatory system, and if complications such as severe bleeding, venous thrombosis, infection, or low blood pressure arise, the patients need knowledge and action competence to reduce complications and secure their safety [3]. For decades, the standard schedule for hemodialysis has been 3 sessions a week, 3-4 hours each time [3]. Chuasuwan et al’s [7] review points out that patients undergoing hemodialysis showed lower QOL than those undergoing PD. Studies indicate a potential survival advantage with intensive dialysis (an increase in dialysis frequency or duration), a goal that can be reached when dialysis takes place in the patient’s home [8]. Due to the high symptom burden and the complexity of their illness and treatment, patients undergoing hemodialysis are dependent on effective and real-time communication with the renal health care professionals for optimal QOL [9]. An increased amount of home hemodialysis (HHD) is an international aim due to patient-centered values of empowerment and QOL. The global increase in CKD poses significant stress on health care systems worldwide, reducing in-center hemodialysis and increasing HHD aimed at reducing both economic burdens and challenges to human resources [10].

Policies and patients’ growing demand for patient participation in health-related decision-making for both in-center hemodialysis and HHD call for new and innovative interventions for eHealth communication [11]. Patient participation is increasingly promoted as a way of making health care more responsive to patient needs and ensuring the legitimacy of decisions affecting patient care [12]. Patient participation has both individual and collective dimensions. This review focuses on the individual dimension that refers to enabling patients to have more influence over their health by increasing their capacity to gain more control over issues they define as important. This review also focuses on the collective dimension that refers to patient participation as collective activities in which patients, relatives, service users, or patient representatives are actively engaged in shaping the development of health care services [13]. Thompson [14] has described patient participation as covering five levels of patient-determined participation or involvement: noninvolvement, given information, dialogue, shared decision-making, and autonomous decision-making. Thompson [14] conceived of professionally determined patient involvement as being along a power continuum from a low level of patient power (exclusion) to a high level of patient power (informed decision-making) in patient consultations. For CKD, patient participation has been offered through technological educational interventions offering patients an opportunity to learn about their condition [15] or self-monitoring aspects of their lifestyle such as nutrition [16]. Patient participation is seen in the technological development and use of standardized shared decision-making interventions (eg, treatment choices) [17]. Such interventions aim to enable patients to be informed, learn about, and engage in their own treatment.

Communication is an essential component of patient engagement and the possibility of autonomy. Responsibility for own patient care, transferred from health personnel to patients, can feel like a scary and overwhelming responsibility instead of strengthening patients’ rights and autonomy. Patients undergoing hemodialysis need to adhere to a strict regimen, and the patients in HHD accordingly handle advanced machines at home and have access to their own blood system, with possibilities of errors and life-threatening complications. Following Thompson’s [14] power continuum when mapping patients’ possibilities for participating in eHealth communication will contribute to defining and stating patients’ self-perceived participation in eHealth dialogue. Whether the alleged contribution from digital solutions provides patients with a feeling of autonomy and flexibility or extra work, burdens, and frustrations can be discussed further throughout the full review.

The World Health Organization defines eHealth as “the cost-effective and secure use of information and communications technologies in support of health and health-related fields, including health-care services, health surveillance, health literature, and health education, knowledge and research” [18]. Sometimes eHealth is referred to as health information technology. Patient participation “requires professionals to engage in two-way communication” [14]. One example of two-way communication is a study evaluating patient and physician perspectives on the key advantages and disadvantages of telephone consultations in a nephrology clinic [19]. Two-way communication might imply the use of computers, mini-computers, and tablets, as well as networks or cloud storage for managing and storing medical records. Mobile health (mHealth) is a narrower concept and refers to the concept of mobile self-care [20], like smartphones and tablet apps used to help people capture data about themselves without assistance or interpretation from health personnel. In recent years, mHealth apps have become increasingly important tools for personalized health care.

eHealth communication is the overarching term used in this review to include communication technologies enabling health professionals and patients to interact, mediated by electronic means [21]. The term incorporates both electronic and digital communication interventions [22]. Many eHealth interventions are multimodal, and definitions may overlap [11]. Examples of communicative eHealth interventions can be related to electronic health records (EHRs) such as care plans. Other examples are patient portals or telehealth solutions such as videoconferencing.

The technological evolution of eHealth communication interventions is claimed to be a paradigm shift for enhanced individuality and patient-centered care, a movement away from the patriarchal traditional health expert–patient relationship. eHealth is predicted to enhance health services and improve care, treatment, effectiveness, and costs, and the development of technology for communication and information sharing has been extensive over the last decades [23]. The COVID-19 pandemic has been a boost for the development and use of eHealth, but the achievement of personalized health care remains an international challenge until there is real data harmonization and interoperability, optimization of data collection, sharing and analytics, and wide acceptance and adoption of innovative digital tools worldwide [23]. Barriers to patients and health professionals participating in eHealth communication could be issues related to the user interface or safety concerns (eg, suboptimal design, technological failure, or invalid data). Facilitators of the use of such technologies are reported to be, for example, systems, programs, education, motivation, patient acceptance and engagement, increased frequency, and quality of interactions with health professionals [11].

### Prior Work

Risling et al [24] intended to explore patient participation in the eHealth context in general but ended up exploring patient portals without focusing on two-way communication. They concluded that measurements for patient participation still need to be operationalized, particularly within eHealth intervention contexts [24].

A preliminary search for existing systematic reviews on the current topic in MEDLINE, the Cochrane Database of Systematic Reviews, and JBI Evidence Synthesis revealed two reviews of the use of mHealth technology in CKD. Schrauben et al [25] have explored CKD patients’ attitudes to mHealth technology in general. Yang et al [26] have explored the use of mHealth technology for patients undergoing dialysis and found that mHealth technology is mostly used for the self-monitoring of, for example, nutrition or diet. The review also shows that patients undergoing hemodialysis are in special need of real-time monitoring and possibilities for prompt feedback [26]. None of the research questions addressed patient participation, barriers and facilitators, or communication. Digital interaction in the EHR between patients living with hemodialysis and health professionals is unsystematically documented [26]. Unique issues relating to involvement in eHealth communication for patients undergoing hemodialysis were scarcely described in either study. Yang et al’s [26] database search ended in October 2018, before the COVID-19 pandemic. The rationale of updating it by means of a scoping review is pertinent, exploring the barriers and facilitators that need to be considered in future interventions. A scoping review methodology is suitable for bringing together literature in disciplines with emerging evidence [27].

### Aims and Review Questions

In this scoping review, we aim to systematize and map emerging research on eHealth communication interventions for patients in hemodialysis and their participative role in the interventions, and to identify barriers and facilitators for participation. Based on a preliminary search for literature, we have found structured literature on, for example, mHealth apps and other one-way eHealth solutions. We have on the contrary found scattered literature on patients’ access to and measurement of their use of eHealth communication interventions as those intending to enable two-way communication between patients undergoing hemodialysis and health professionals. Therefore we find it relevant to search systematically and broadly. All three authors have clinical experience in nursing and research, and have contributed with ideas and further development of the review questions. The theme and questions have been approved by two user representatives from the Norwegian interest group The Norwegian Kidney Patients Association (Landsforeningen for Nyresyke og Transplanterte). The findings of this scoping review will inspire a process and further studies to develop eHealth communication between patients and nurses in hemodialysis care.

The review will be guided by the following research questions:

- Which type of eHealth communication interventions for patients undergoing hemodialysis and health professionals can be identified in the literature and how are they linked to the EHR?
- Which participative role for patients undergoing hemodialysis in eHealth communication with health professionals can be identified in the literature?
- What are the key participative barriers and facilitators to eHealth communication with health professionals encountered by patients undergoing hemodialysis?

## Methods

### Overview

In this protocol, we predefine the objectives, research questions, and methods, and detail the proposed plan for a scoping review. The proposed scoping review will be conducted according to the Joanna Briggs Institute methodology for scoping reviews [27] and reported following the PRISMA-ScR (Preferred Reporting Items for Systematic Reviews and Meta-Analyses Extension for Scoping Reviews) [28].

This protocol was developed in line with the abovementioned methodology and was reported according to the PRISMA-P (Preferred Reporting Items for Systematic Review and Meta-Analysis Protocols) checklist [29].

### Eligibility Criteria

We used the PCC (Population, Concept, and Context) mnemonic [27] to construct the review and define inclusion and exclusion criteria.

#### Population

This review will consider studies that include adult patients (18 years or older) with CKD5 and undergoing hemodialysis. All types of hemodialysis are covered, including prescheduled in-center dialysis, conventional HHD, short daily HHD, and nocturnal HHD. Studies focusing solely on people younger than 18 years, on people living with CKD stages 1-4, on kidney transplants, and on people undergoing PD will be excluded. Studies including these people will be included if they also include patients undergoing hemodialysis.

#### Concept

This review will consider studies that explore a three-folded concept.

First, we will use a wide definition for interventions. In this review, intervention is a collective term for concepts including, but not limited to, actions, processes, measures, strategies, and initiatives to develop eHealth communication [22]. We will consider all types of eHealth communication interventions or solutions intended to allow two-way communication between patients and health professionals. In this review, we included both electronic and digital technology for oral or written communications (eg, EHRs), including standardized nursing terminology, electronic patient records or portals, electronic conferencing, and mobile written or oral communication mediated by electronic or digital means. Studies on eHealth interventions with no possibility for communication between patients with CKD and health professionals will be excluded (eg, mobile apps for self-efficacy).

Second, patient participation in this review refers to Thompson’s [14] definition, stating that patient participation “requires professionals to engage in two-way communication.” We will consider the inclusion of studies on all levels of the continuum of patient participation, involvement, and similar concepts.

Third, barriers concerning patient participation in eHealth communication may include but are not restricted to problems, issues, challenges, or obstacles to participation in eHealth communication. Facilitators of patient participation may include but are not restricted to recommendations, interventions or programs, motivation, or experienced results [11].

#### Context

This review will consider studies focusing on in-center hemodialysis (hospital and satellite units) and HHD contexts.

### Information Sources

With no limits on the publication date, we will perform a systematic search for English language articles in the following electronic databases: MEDLINE, Embase, CINAHL, Scopus, and ProQuest Dissertations and Theses Open. We will consider published peer-reviewed primary studies, including quantitative, qualitative, and mixed methods study designs. We will also consider relevant dissertations and theses. Conference abstracts, unpublished studies, literature reviews, and Master’s theses will be excluded.

### Search Strategy

First, we performed an initial limited search of MEDLINE (Ovid) and CINAHL (EBSCO) to identify articles and search terms on the topic. Text words contained in the titles and abstracts of relevant articles, and the index terms used to describe the articles have been used to develop a full search strategy for MEDLINE (see Multimedia Appendix 1). The search strategy was structured according to PCC (ie, patients undergoing hemodialysis, eHealth interventions with the possibility for written or oral communication, and patient participation). A librarian assisted in developing the search strategy based on the PRESS (Peer Review Electronic Search Strategies) guidelines [30].

Second, the search strategy, including all identified keywords and index terms, will be adapted for each included database. These databases have been discussed and chosen by the authors and the librarian to ensure that a broad overview of published literature within our field will be retrieved by our search. The librarian will assist the search process [30].

Third, the reference list of articles included in the review will be manually searched for additional studies of relevance.

If both a peer-reviewed article and a dissertation are available concerning the same study, we will prioritize the peer-reviewed publication for data extraction. Authors of papers will be contacted to request missing or additional data if required.

### Data Management

Following the search, all identified records will be collated and uploaded into the reference management tool EndNote 20 (Clarivate Analytics). Duplicates will be removed. The screening software tool Rayyan (Qatar Computing Research Institute) will be used to facilitate the screening process.

### Study Selection

Initially, a pilot test will be trialed by the review team to ensure consensus on which articles are considered to meet the inclusion and exclusion criteria. A total of 25 randomly selected articles will be screened by titles and abstracts individually by two reviewers (AD and CFM) and discussed with the third reviewer. Once consensus has been reached, all titles and abstracts will be screened independently by two reviewers for assessment against the inclusion criteria. Potential studies for inclusion will be read in the full text, and studies not meeting the inclusion criteria will be reported with the reason for exclusion. This scoping review is not aimed at making practice recommendations; it rather seeks to provide an overview of the collected data rather than synthesize the evidence [26]. Therefore, methodological quality appraisal of included studies is not pertinent. Any disagreements that arise between the two reviewers at each stage of the selection process will be resolved based on consensus through discussion or with the third reviewer (MSL). The results of the search and the study inclusion process will be detailed and reported in full in the final scoping review and presented in a PRISMA flow diagram [31].

### Data Extraction and Collection

A draft charting table (Multimedia Appendix 2) has been developed at this protocol stage by the reviewers, and the data extraction tool will be piloted. The pair of authors (AD and CFM) will individually conduct the data extraction. The data extracted will include specific details about the population, concept, context, methods, and key findings of included articles. Relevant to the review questions, the type of eHealth intervention will be extracted and systemized, and any linking to the EHR will be mapped. The type of participatory role will be determined according to Thompson’s [14] taxonomy, and data will be related to the five levels of patient participation described in the taxonomy.

To provide an overview of the collected data, we will use a basic descriptive qualitative coding to identify barriers and facilitators related to participation in eHealth communication interventions [27]. The method of data analysis will also be used to develop and present a narrative description of the data [32].

The draft charting table will be modified and revised as necessary during the process of extracting data from each included paper. Modifications will be detailed in the full scoping review. Any disagreements that arise between the reviewers will be resolved through discussion or with the third reviewer.

## Results

The extracted data will be presented in diagrammatic or tabular form in a manner aligned with the objective of the scoping review. The report will also include a narrative summary to accompany the tabulated or charted results. We will present findings on the participative role of patients undergoing hemodialysis in eHealth communication interventions. The collated results will be presented in a systematic scoping review publication related to the review’s objective and questions, which is planned to be submitted in the spring of 2023.

## Discussion

### Contribution to eHealth Communication Development

The anticipated main findings of this study would be a presentation of the types of eHealth communication interventions and patients’ experiences with their use of them. A strength of this study is the systematic, precise, and comprehensive process of working out the search strategy in cooperation with the librarian. The rapid and extensive development of eHealth interventions, which is claiming to solve future communication challenges, needs participation from those involved [11]. The anticipated results are expected to show the barriers and facilitators to patient participation and the levels of patient participation.

### Conclusions

We are focusing on patient rights by mapping the participative patient role, which can contribute to a further focus on patient participation. The findings can identify research gaps of importance to future research, the development of communicative eHealth interventions, and clinical practice. The systematized knowledge can be transferable to other patient groups, health personnel, researchers, and decision makers in future cocreating processes. The findings from the scoping review can be included as part of the knowledge base for further development of eHealth communication solutions. The knowledge acquired can contribute to developing eHealth interventions to strengthen the relations between service users and service providers. The results will be shared with key stakeholders in the development, use, and policies of eHealth interventions.

## Appendix

Multimedia Appendix 1 Search strategy MEDLINE.

Multimedia Appendix 2 Draft charting table.

## Abbreviations

CKD: chronic kidney disease
EHR: electronic health record
GFR: glomerular filtration rate
HHD: home hemodialysis
mHealth: mobile health
PCC: Population, Concept, and Context
PD: peritoneal dialysis
PRESS: Peer Review Electronic Search Strategies
PRISMA-P: Preferred Reporting Items for Systematic Review and Meta-Analysis Protocols
PRISMA-ScR: Preferred Reporting Items for Systematic Reviews and Meta-Analyses Extension for Scoping Reviews
QOL: quality of life
