# Supplementary material for: Interventions, Participative Role, Barriers, and Facilitators for Involvement in eHealth Communication for People Undergoing Hemodialysis: Protocol for a Scoping Review
Source: JMIR Res Protoc. 2022 Jul 29;11(7):e38615. doi: 10.2196/38615 (PMC9377479; doi:10.2196/38615)
Supplement: Multimedia Appendix 1 [file resprot_v11i7e38615_app1.docx]

**Appendix I: Search strategy MEDLINE**

MEDLINE (Ovid) Date searched: 06.04.22, 139 Records retrieved

| Search | Query | Records retrieved |
| --- | --- | --- |
| #1  MeSH and textwords (TW) | Hemodialysis Units, Hospital/ or Renal Dialysis/ or Hemodialysis, Home/ or Renal Insufficiency, Chronic/ or Kidney Failure, Chronic/ or (h?emodialys* or ((extracorporeal or renal) adj dialys?s) or ((chronic or end stage or endstage) adj (kidney or renal) adj (disease? or failure or insufficienc*)) or (CKD or ESRD) or (home adj (dialys?s or h?emodialys*)) or ((dialys?s or h?emodialys*) adj (cent* or department? or unit?))).tw,kf. | 232864 |
| #2  MeSH and TW | Decision Making, Shared/ or Patient Access to Records/ or Patient Reported Outcome Measures/ or Patient Outcome Assessment/ or Patient Participation/ or Consumer Health Information/ or Health Literacy/ or ((shared adj2 decision?) or (patient? access to adj2 (record? or data)) or ((patient-reported or self-reported) adj outcome?) or PROM or patient outcome assessment? or (patient adj2 (activat* or empower* or engag* or involv* or participat* or report*)) or consumer health information or (health adj2 literacy) or (co-creation or cocreation) or ((consumer or user) adj participation)).tw,kf. | 137798 |
| #3  MeSH and TW | Telemedicine/ or Telenursing/ or Medical Records/ or Health Records, Personal/ or Patient Portals/ or exp Electronic Health Records/ or Nursing Records/ or Medical Informatics Applications/ or Consumer Health Informatics/ or Mobile Applications/ or Smartphone/ or Cell Phone/ or Computers, Handheld/ or Health information management/ or Standardized Nursing Terminology/ or (mobile health or telehealth or tele-health or telemedicine or tele-medicine or telenursing or tele-nursing or (eHealth or e-health or e-communication or mHealth or m-health) or (tablet? or smartphone? or smart phone? or mobile phone? or cellular phone? or cellphone? or cell phone?) or (mobile adj2 (app? or application? or device?)) or ((medical or nursing or health) adj record?) or electronic health record? or (EPR or EHR or EPHR) or (patient adj2 (portal? or record?)) or ((medical or health) adj information adj (exchange? or technolog* or record? or management?)) or health informatic? or ((digital or structured) adj decision adj tool?) or health diar* or personal health information or medical informatics application? or nursing terminolog*).tw,kf. | 361862 |
| #4 | #1 AND #2 AND #3 | 139 |
| Limited to English language | | |

* The participants and the context search terms are mutually inclusive. The concept search terms are divided in two search blocks.

Searched: #1 Population, Context, MeSH + TW

#2 Concept 1, MeSH + TW

#3 Concept 2, MeSH + TW

#4 #1 AND #2 AND #3 Records retrieved: 139
