# Supplementary material for: Interventions, Participative Role, Barriers, and Facilitators for Involvement in eHealth Communication for People Undergoing Hemodialysis: Protocol for a Scoping Review
Source: JMIR Res Protoc. 2022 Jul 29;11(7):e38615. doi: 10.2196/38615 (PMC9377479; doi:10.2196/38615)
Supplement: Multimedia Appendix 2 [file resprot_v11i7e38615_app2.docx]

**Appendix II: Draft charting table**

| Authors(s) |  |
| --- | --- |
| Year of publication |  |
| Title, Journal (volume, issue, pages) |  |
| Country of origin |  |
| Aim(s) of article |  |
| Method(s) and study design |  |
| Participants/ population (age)  Hospital- or home hemodialysis |  |
| Type of eHealth-communication intervention used |  |
| The intervention`s link/ connection to electronic health record |  |
| Type/ level of patient participation according to Thompson. *Thompson AGH. The meaning of patient involvement and participation in health care consultations: A taxonomy. Soc Sci Med. 2007; 64(6):1297-310* |  |
| Barriers encountered by patients |  |
| Facilitators encountered by patients |  |
| HD-context (home-, satellite-, -unit, -hospital) |  |
